# INVESTIGATING MODERATORS OF THE RELATIONSHIP BETWEEN SUBJECTIVE AND OBJECTIVE MEMORY

**DOI:** 10.1093/geroni/igz038.158

**Published:** 2019-11-08

**Authors:** Francesca Falzarano, Jillian Minahan, Karen L Siedlecki, Timothy Salthouse

**Affiliations:** 1 Fordham University, Bronx, New York, United States; 2 University of Virginia, Charlottesville, Virginia, United States

## Abstract

Subjective memory complaints (SMC) among older adults have been explored as an indicator of decline in objective memory functioning. While some research has found that SMC may be predictive of future cognitive impairment and dementia (Glodzik-Sobanska et al., 2007; Wang et al., 2004), others have suggested that SMC are common among healthy older adults (Cooper et al., 2011) and are not strongly related to objective memory performance. Researchers suggest that SMC may be more strongly related to affective factors (e.g., depression and anxiety; Rowell, Green, Teachman, & Salthouse, 2015). The current study examined the relationship between SMC, objective episodic memory performance (OEMP), along with depression and anxiety in a sample of 18-99 year olds (N = 5,430) from the Virginia Cognitive Aging Project (VCAP). Structural equation modeling with full information maximum likelihood estimation was used to investigate whether clinically-relevant depression and anxiety levels moderated the relationship between SMC and OEMP, controlling for age, education, gender, and health. OEMP was represented as a latent construct while the remaining variables were observed. Although depression and anxiety are significantly related to SMC (r’s = .29, .17, respectively), they are not correlated with OEMP. Furthermore, depression, but not anxiety, moderated the relationship between SMC and OEMP, such that those at risk for depression had a stronger relationship between SMC and OEMP (-.07, p<.05) compared to those not at risk (-.02, p=.31). This suggests that SMC may not be a valid indicator of OEMP as it may reflect variance from other sources, such as depression.

